# *TLCD4* as Potential Transcriptomic Biomarker of Cold Exposure

**DOI:** 10.3390/biom14080935

**Published:** 2024-08-01

**Authors:** Bàrbara Reynés, Estefanía García-Ruiz, Evert M. van Schothorst, Jaap Keijer, Paula Oliver, Andreu Palou

**Affiliations:** 1 Nutrigenomics, Biomarkers and Risk Evaluation (NuBE) Group, University of the Balearic Islands, 07122 Palma, Spain; barbara.reynes@uib.es (B.R.);; 2 Health Research Institute of the Balearic Islands (IdISBa), 07120 Palma, Spain; 3 CIBER of Physiopathology of Obesity and Nutrition (CIBEROBN), Instituto de Salud Carlos III, 28029 Madrid, Spain; 4 Human and Animal Physiology, Wageningen University, 6708 WD Wageningen, The Netherlands; evert.vanschothorst@wur.nl (E.M.v.S.);

**Keywords:** cold exposure, biomarker, thermogenesis, browning, PBMC, adipose tissue

## Abstract

(1) Background: Cold exposure induces metabolic adaptations that can promote health benefits, including increased energy disposal due to lipid mobilization in adipose tissue (AT). This study aims to identify easily measurable biomarkers mirroring the effect of cold exposure on AT. (2) Methods: Transcriptomic analysis was performed in peripheral blood mononuclear cells (PBMCs) and distinct AT depots of two animal models (ferrets and rats) exposed to cold, and in PBMCs of cold-exposed humans. (3) Results: One week of cold exposure (at 4 °C) affected different metabolic pathways and gene expression in the AT of ferrets, an animal model with an AT more similar to humans than that of rodents. However, only one gene, *Tlcd4*, was affected in the same way (overexpressed) in aortic perivascular and inguinal AT depots and in PBMCs, making it a potential biomarker of interest. Subsequent targeted analysis in rats showed that 1 week at 4 °C also induced *Tlcd4* expression in brown AT and PBMCs, while 1 h at 4 °C resulted in reduced *Tlcd4* mRNA levels in retroperitoneal white AT. In humans, no clear effects were observed. Nevertheless, decreased PBMC *TLCD4* expression was observed after acute cold exposure in women with normal weight, although this effect could be attributed to short-term fasting during the procedure. No effect was evident in women with overweight or in normal-weight men. (4) Conclusions: Our results obtained for different species point toward *TLCD4* gene expression as a potential biomarker of cold exposure/fat mobilization that could tentatively be used to address the effectiveness of cold exposure-mimicking therapies.

## 1. Introduction

Exposure to cold could have health benefits and be used as a therapeutic strategy for obesity and its comorbidities, such as type 2 diabetes, mainly due to its effects on adipose tissue remodeling [1]. Cold exposure induces brown adipose tissue (BAT) recruitment and activation as well as the appearance of brown-like or beige adipocytes [2]. The latter are characterized by a high content of mitochondria that express uncoupling protein 1 (UCP1) and by the presence of multilocular lipid droplets. This type of adipocyte resides within white adipose tissue (WAT) and is thermogenically inducible in a process known as browning [3]. BAT thermogenesis and WAT browning have been associated with increased energy expenditure, improved lipid, and glucose metabolism, and improved metabolic health both in rodents and humans [4]. 

In adult humans, brown fat has been detected using (18)F-fluorodeoxyglucose ((18)F-FDG) positron emission tomographic and computed tomography (PET-CT). This fat is distributed mostly around the cervical and supraclavicular, parasternal, and sometimes perirenal regions [4,5,6]. Molecular analyses performed by different authors have revealed that human brown fat depots present both brown and beige adipocytes [7,8,9,10]. Thus, though more studies are needed, as the induction of WAT browning in humans could be a promising tool for anti-obesity treatments. 

Most human thermogenic studies are based on 18F-FDG PET analysis or on transcriptomic analysis performed in adipose tissue biopsies [11], which are invasive techniques. The presence of another biological material that is more easily obtainable would improve thermogenic/browning studies on healthy humans. In this line of thinking, our previous studies performed on rodents revealed that peripheral blood mononuclear cells (PBMCs) can express brown/beige markers and reflect BAT activation and WAT browning in response to cold exposure and to the intake of high-fat diets [12,13]. More recently, Efremova et al. pointed out that PBMCs of healthy miners from Siberia express the brown adipocyte marker *CIDEA* and the browning marker *HOXC9*, whose expression varies with cold exposure, possibly reflecting a thermogenic response [14].

With this background, our main aim was to identify cold exposure transcriptomic biomarkers related to cold exposure using a minimally invasive material, to facilitate human studies. For this purpose, we performed a global transcriptomic analysis in PBMCs and different adipose tissue depots of longer-term cold-exposed ferrets. This animal model was selected because of the closer similarity of their adipose tissue to that of humans, in comparison to rodents [15]. Afterward, a selected transcriptomic biomarker of cold response was validated in PBMCs and different adipose tissues of cold-exposed rats, and in PBMCs of cold-exposed humans, also analyzing the effect of acute and longer-term exposure to cold.

## 2. Materials and Methods

### 2.1. Experimental Designs

This study used animal models (ferrets and rodents) as well as humans. All the animal protocols were in accordance with the university’s ethical guidelines for the use and care of laboratory animals and were approved by the Ethics Committee of Bioethics Commission of the University of the Balearic Islands (registration number 9207, approval date: 21 July 2011). All animals were exposed to a light/dark cycle of 12 h and had free access to water and diet; when cold-exposed, animals were housed individually. The human study was approved by the Ethics Committee of Research of the Balearic Islands (CEI-IB) (Approval Code IB 3146/16 PI, approval date: 16 May 2016) and was carried out in accordance with The Code of Ethics of the World Medical Association (Declaration of Helsinki). Informed written consent was obtained from all participants. The recruitment of the study subjects and data/sample collection were conducted in Mallorca from July 2016 to July 2018.

#### 2.1.1. Ferret Experimental Design

Ferrets were selected because their AT has a closer resemblance to the AT of humans compared to rodents [15]. Thus, male ferrets (*Mustela Putorius Furo* from Cunipic, Lleida, Spain) of three months of age, fed with a control diet (Gonzalo Zaragoza Manresa SL, Alicante, Spain), were divided into two groups: a control group (*n* = 7), acclimatized to room temperature (22 ± 2 °C); and a cold group (*n* = 6), acclimatized to 4 °C for one week. At the end of the experimental period, ferrets were weighed and killed by exsanguination. The animals were anesthetized using 10 mg/kg of ketamine hydrochloride (Imalgène 1000, Merial Laboratorios SA, Lyon, France) and 80 mg/kg medetomidine (Domtor, Orion Pharma, Espoo, Finland). Arterial blood was collected from the left ventricle using heparin in NaCl (0.9%) as an anticoagulant. Afterward, different adipose depots (aortic perivascular—aPVAT—and inguinal–IAT–) were rapidly removed and weighed, frozen in liquid nitrogen, and stored at −80 °C until RNA analysis. 

#### 2.1.2. Wistar Rat Experimental Designs

Two experimental designs, longer-term and acute cold exposure, were performed with female rats. Female rats were selected because they seem to be more sensitive to longer-term cold exposure than males, as reviewed in [16]. In Experiment 1 (longer-term cold exposure), female Wistar rats (Charles River Laboratories España, Barcelona, Spain) of different ages (1, 2, 4, and 6 months), were divided into two groups: a control group (*n* = 6, except at *n* = 5 for 4 months of age), acclimatized to room temperature (22 ± 2 °C) to mimic human physiology [17]; and a cold group (*n* = 6), acclimatized to 4 °C. Both of them were exposed for one week (longer-term cold exposure). Different ages were used because age is known to affect the cold response, which appears to be greater in young animals [12]. In Experiment 2 (acute cold exposure), we used female Wistar rats (Charles River Laboratories España, Barcelona, Spain) of one month of age due to their higher sensitivity to cold, divided into three groups (n = 8 per group): a control group, acclimatized to room temperature (22 ± 2 °C); and two cold groups acclimatized to 4 °C during one or two hours (acute cold exposure). In this second experiment, we used acute cold exposure (1–2 h) to make it more comparable to the human situation, as human cold-exposure studies use mainly a few hours of cold acclimation [18,19,20,21]. 

All Wistar rats were fed ad libitum with a standard chow diet (Panlab, Barcelona, Spain). At the end of the experimental period, the rats were weighed and killed by decapitation, and blood was collected from the neck with EDTA for PBMC isolation. Afterward, visceral (retroperitoneal) and subcutaneous (inguinal) WAT depots and interscapular BAT were rapidly removed and weighed, frozen in liquid nitrogen, and stored at −80 °C until RNA analysis. 

#### 2.1.3. Human Experiment

The cold exposure response is blunted by overweight [18], and the thermogenic capacity seems to be affected by sex [16]. In this sense, to evaluate the suitability of *TLCD4* (the biomarker of interest identified as the result of the ferret and rat experiments) as a transcriptomic indicator of cold exposure, a group of women and men presenting normal weight, and a group of women with overweight were analyzed. All in all, fourteen young women and seven young men were recruited. Women were classified into two groups depending on their body mass index (BMI): normal weight (*n* = 8) with BMI < 25 kg/m^2^, and women with overweight or obesity (*n* = 6) with BMI ≥ 25 kg/m^2^. All men were normal weight (*n* = 6) with BMI <25 kg/m^2^. The inclusion criteria were as follows: subjects aged 18–30 years old, without metabolic diseases, keeping their usual diet, and without a legal or physical inability. All subjects followed the same protocol: after 1 h of acclimatization to room temperature (22 ± 2 °C), the participants spent 2 h in a room with a temperature of 17 ± 1 °C, with an intermittent immersion (5 min in/5 min out) of feet in cold water (approximately 8 ± 2 °C) while wearing light clothing. These conditions were selected as different studies have shown that 17 °C cool air exposure for 2 h, in combination with the feet placed intermittently in ice water, induces fluoro-glucose uptake rate in humans, revealing the increased prevalence of BAT in humans [18,20,21]. Subjects were in fasted conditions for 4 h; anthropometric parameters (body weight, height, waist and hip perimeter, BMI, and % of fat) were collected (data are available in Supplementary Table S1), and then, blood samples were collected before (Point 1) and after two hours of cold exposure (Point 2). The study was repeated without cold exposure as the control.

### 2.2. Isolation of Peripheral Blood Mononuclear Cells

Ferret PBMCs were isolated from sampled blood by Ficoll gradient separation according to the instructions of the manufacturer (GE Healthcare Bio Sciences, Barcelona, Spain), with some modifications [22]. Briefly, the anticoagulant-treated blood was diluted with an equal volume of balanced salt solution, which was prepared by mixing two stock solutions (1/10): solution A (5.5 mM anhydrous D-glucose, 5 mM CaCl_2_·2H_2_O, 0.98 mM MgCl_2_·6H_2_O, 5.4 mM KCl, 145 mM Tris) and solution B (140 mM NaCl). Afterward, the blood was layered carefully over Ficoll in a centrifuge tube, without intermixing, and centrifuged at 900× *g* for 40 min at 20 °C, with the acceleration and deceleration adjusted at zero. The PBMC-containing interface was harvested from the tub, washed with the previously described balanced salt solution, and centrifuged at 400× *g* for 10 min at 20 °C. Rat PBMCs were isolated using OptiPrep (Sigma-Aldrich Química, SL, Madrid, Spain) gradient separation [12]. In brief, solution C (146 mM NaCl and 1 mM HEPES, with adjusted pH 7.4) was added to EDTA-treated blood samples to a final volume of 6 mL. Blood samples in solution C were layered over the density barrier (2.7 mL OptiPrep and 9.3 mL OptiPep diluent, composed of Solution C diluted 1/1.22 in water) without intermixing. Then, the samples were centrifuged at 700× *g* for 20 min at 20 °C, with the acceleration and deceleration adjusted to zero. The PBMCs and platelets layer were harvested and washed with solution C. This material was then centrifuged in Solution C at 400× *g* for 10 min at 20 °C to wash isolated PBMCs. Human PBMCs were isolated by diluting EDTA anticoagulated venous blood from participants 1:1 with PBS1X. The mix was layered carefully over Ficoll (GE Healthcare BioScience, Barcelona, Spain) in a centrifuge tube, which was centrifuged for 30 min at 400× *g* at 20 °C, with the acceleration and deceleration adjusted to zero. The PBMC layer was collected and centrifuged in PBS1X at 300× *g* for 10 min at 20 °C to wash isolated PBMCs.

### 2.3. Total RNA Isolation

In the ferret experiment, total RNA was extracted using TriPure Reagent (Roche Diagnostics Barcelona, Spain) and then purified with E.Z.N.A. MicroElute RNA Clean Up (Omega Bio-Tek, Winooski, VT, USA) and via precipitation with 3M sodium acetate and absolute ethanol. Total RNA from rat samples was extracted using TriPure Reagent (Roche). Finally, isolated RNA from all tissue samples was purified via precipitation with 3M sodium acetate and absolute ethanol. Concerning the human experiment, total RNA from human PBMC samples was extracted using TriPure Reagent (Roche). The RNA yield from the different experimental designs was quantified on a NanoDrop ND 1000 spectrophotometer (NanoDrop Technologies, Wilmington, DE, USA).

### 2.4. Microarray Processing

We used an Agilent array custom-designed for our laboratory in collaboration with the Príncipe Felipe Research Centre. The ferret array encompassed 45,328 sequences, encoding a total of 19,299 unique genes. aPVAT, IAT, and PBMC RNA samples of ferrets of the control (*n* = 7, except for PBMCs in which only 6 RNA samples were used due to difficulties in obtaining sufficient RNA from PBMCs of one of the animals) and cold groups (*n* = 6, except for PBMCs in which only 4 RNA samples were used due to not having enough RNA from two of the animals) were used. The microarray procedure and analysis were performed and normalized as previously described [22]. Statistical differences between the cold-exposed group vs. the control group were assessed using a Student’s *t*-test in GeneMaths XT 2.12; the generated *p*-values were used to obtain insight into significantly affected genes. Fold change calculations were performed in Microsoft Excel (version 16.87). The common DEGs (Student’s *t*-test *p* < 0.05) from aPVAT, IAT, and PBMCs were manually classified into biological processes using available databases (Genecards, NCBI, WikiPathways, PubMed), focusing on key biological domains, such as molecular function and biological processes. The microarray chip presented different sequences for each gene; the analysis was performed independently of the sequence, only considering the genes. Moreover, the common DEGs with a more restrictive *p*-value were also studied (Student’s *t*-test *p* < 0.01). To control for false positives, we performed the Benjamini–Hochberg false discovery rate (FDR) test, and all the sequences of the unique gene up-regulated in the two adipose tissues studied and in PBMCs, have an adjusted *p*-value < 0.39. Microarray data were deposited in the NCBI Gene Expression Omnibus (GEO) under accession number GSE62353 for aPVAT, GSE62351 for IAT, and GSE62352 for PBMC datasets.

### 2.5. Real-Time Reverse Transcriptase Polymerase Chain Reaction (RT-qPCR) Analysis

Fifty ng of total RNA from ferret aPVAT, IAT, and PBMCs, as well as from the PBMCs of the human and rat experiments were reverse transcribed to cDNA using a iScript cDNA synthesis kit (BIO-RAD, Madrid, Spain) at 25 °C for 5 min, 42 °C for 30 min, and 85 °C for 5 min in an Applied Biosystems 2720 Thermal Cycler (Applied Biosystems, Madrid, Spain). Otherwise, 250 ng of total RNA from rat adipose tissue samples were denatured and then reverse transcribed to cDNA using Multireverse transcriptase (Applied Biosystem) as previously described [12]. Each PCR was performed from 1/10 diluted cDNA for adipose tissue samples or 1/5 for PBMCs, forward and reverse primers (5 µM), and Power SYBER Green PCR Master Mix (Applied Biosystems) in a total volume of 11 µL, with the following profile: 10 min at 95 °C, followed by a total of 40 temperature cycles (15 s at 95 °C and 1 min at 60–62 °C) with a final cycle of 15 s at 95 °C, 1 min at 60 °C, and 15 s at 95 °C. The threshold cycle (Ct) was calculated using the instrument’s software (StepOne Software v2.0, Applied Biosystems) and the relative expression of each mRNA was calculated as a percentage of control animals, using the 2^−ΔΔCt^ method [23]. Data were normalized against housekeeping genes. For the ferret experiment, we selected *Methyltransferase like 2B* (*Mettl2b*), which has been revealed as a stable reference gene for cold exposure in ferret samples in the microarray analysis performed. For rats, *Low-density lipoprotein receptor-related protein 10* (*Lrp10*) and *Guanosine Diphosphate Dissociation Inhibitor 1* (*Gdi*), two well-known reference genes were selected. For humans, *Ribosomal protein, large, P0* (*RPLP0*), another well-known reference gene was used. All primers, described in Supplementary Table S2, were obtained from Sigma Genosys (Sigma Aldrich Química SA, Madrid, Spain).

### 2.6. Statistical Analysis

All data are expressed as the mean ± SEM. The normal distribution of the data and homogeneity of variances were tested using the Shapiro–Wilk test and the Levene test, respectively. Differences between two groups were analyzed using the Student’s *t*-test for parametric data, while the Mann–Whitney U test and Wilcoxon test were used for non-parametric data. Differences between different times of cold exposure were analyzed using one-way ANOVA. A LSD post hoc test was used after ANOVA analysis. The data that did not meet the required rules for one-way ANOVA were log10-transformed. The specific statistical analysis used for each comparison is specified in the footnotes of the figures. The threshold of significance was defined at *p* < 0.05, and it is indicated when different. The analyses were performed with SPSS for Windows (SPSS, Chicago, IL, USA). Statistics used for the microarray data are described in the Section 2.4.

## 3. Results

### 3.1. Body Weight and Adiposity Parameters in Animals after Longer-Term Cold Exposure

Body weight and adiposity data of the same cohorts of ferrets and rats used in the present manuscript have been previously published [12,22]. Briefly, in ferrets, one-week cold exposure decreased the size of interscapular, inguinal, and retroperitoneal adipose tissues although body weight was not significantly affected [22]. In rats, one-week cold exposure decreased adiposity at the different ages studied (1, 2, 4, and 6 months). A decrease in body weight was only observed in the one- and four-month-old animals [12].

### 3.2. Microarray Analysis in Adipose Tissue and PBMC of Cold-Exposed Ferrets

**Figure 1:**
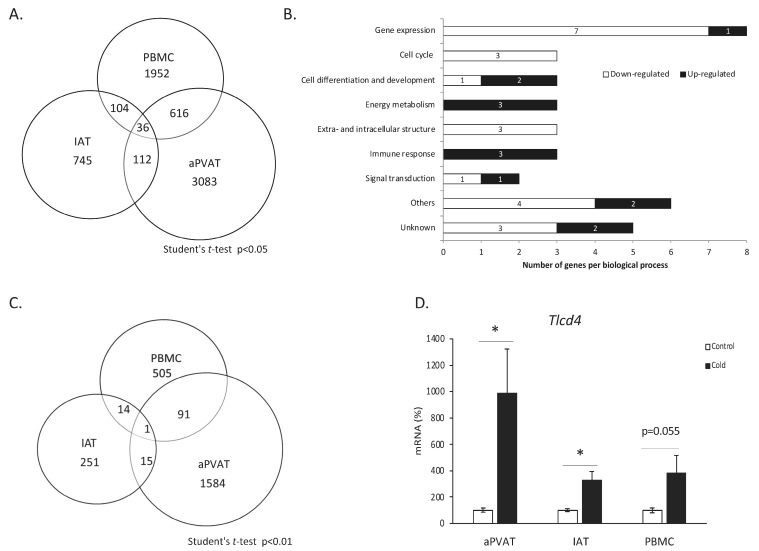
Schematic overview of regulated genes in aPVAT, IAT, and PBMCs in control (22 °C) vs. one-week cold-exposed (4 °C) male ferrets. The overlapping genes between aPVAT, IAT, and PBMCs are those equally regulated. Student’s *t*-test, *p*-value < 0.05 (**A**) or *p*-value < 0.01 (**C**). The detailed manual classification of the 36 genes equally regulated in the three types of samples (Student’s *t*-test, *p*-value < 0.05) (**B**). *TLCD4* gene expression in aPVAT, IAT, and PBMCs of the same set of ferrets (**D**). The mRNA expression was measured by real-time RT-qPCR. The results represent the means ± SEM (*n* = 4–7) of the ratios of specific mRNA levels relative to *Mettl2b*, expressed as a percentage, where the control group was set as 100%. Statistics: the * symbol shows the significance of the cold-exposed groups vs. the control group (U Mann–Whitney, *p* < 0.05, or indicated when different).

Global gene expression was assessed by microarray analysis in aPVAT, IAT, and PBMCs from ferrets housed at 22 °C or exposed to 4 °C for one week to obtain transcriptomic biomarkers of cold exposure. Like humans, ferrets do not possess a well-defined BAT, but their adipose tissue has browning capacity [15]. The tissue with a more pronounced gene expression response to cold was aPVAT. A total of 3847 differentially expressed genes (DEGs) were identified between cold-exposed and control ferrets. In the PBMCs, 2708 DEGs were found, and 997 in IAT (Student’s *t*-test, *p*-value < 0.05) (Figure 1A). Of these, 652 DEGs were affected in the same way between aPVAT and PBMCs, 148 between aPVAT and IAT, and 140 between IAT and PBMCs. The number of DEGs similarly affected by cold exposure in the three types of samples was just 36. These 36 commonly regulated genes were manually classified into the following biological processes: gene expression, cell cycle, cell differentiation and development, energy metabolism, extra and intracellular structure, immune response, signal transduction, and others or unknown (Student’s *t*-test, *p*-value < 0.05) (Figure 1B). With a more restrictive *p*-value (Student’s *t*-test, *p*-value < 0.01), 1691 DEGs were identified by cold exposure in aPVAT, 611 in PBMCs, and 281 in IAT (Figure 1C). aPVAT and PBMCs had 92 common DEGs that responded in the same manner. There were 16 common DEGs between aPVAT and IAT, and 15 between IAT and PBMCs. Interestingly, with this more restrictive *p*-value, only one gene was affected by cold exposure in the same way in the PBMCs, aPVAT, and IAT. This gene is *TLC domain containing 4* (*Tlcd4*), whose expression was increased by cold exposure in the two adipose tissues analyzed, as well as in the PBMCs. Moreover, it should be pointed out that in IAT, *Tlcd4* was one of the top five regulated sequences by cold exposure, according to its *p*-value.

### 3.3. TLCD4 Gene Expression in Response to Cold Exposure

To validate the microarray analysis, the *Tlcd4* gene expression was assessed by RT-qPCR. In ferrets, aPVAT, IAT, and PBMC total RNA samples were used and, in all cases, an increased expression was observed in the cold-exposed group, confirming the microarray data (Figure 1D). 

To confirm whether *Tlcd4* is a good transcriptomic biomarker of cold exposure, we also measured its gene expression in rodents. Specifically, female rats were used because most of the published studies indicate that female rats seem to be more sensitive to cold than male rats in terms of thermogenic response [16].

**Figure 2:**
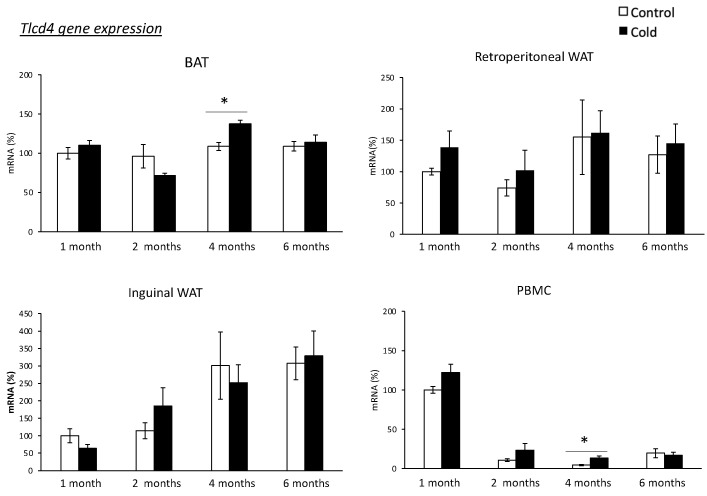
*Tlcd4* gene expression in the inguinal and retroperitoneal WAT, in BAT and PBMCs of female rats of different ages (from 1 to 6 months) housed at different room temperatures: 22 °C (Control) or 4 °C for one week (Cold). mRNA expression was measured by real-time RT-qPCR. Results represent means ± SEM (*n* = 4–6) of ratios of specific mRNA levels relative to *Lrp10*, expressed as a percentage of the value of 1-month-old control animals that was set to 100%. Statistics: the * symbol shows the significance of the cold-exposed groups vs. the control group (U Mann–Whitney, *p* < 0.05, or indicated when different).

First, a longer-term cold-exposure (one week at 4 °C) experiment was performed with rats of different ages. As shown in Figure 2, the BAT *Tlcd4* mRNA levels increased with one-week cold exposure in 4-month-old rats (Mann–Whitney U test, *p* < 0.05). The same pattern was observed in PBMCs of cold-exposed rats, where higher *Tlcd4* mRNA expression levels were observed at the age of 4 months (Mann–Whitney U test, *p* < 0.05). No significant results were obtained for the other ages analyzed.

**Figure 3:**
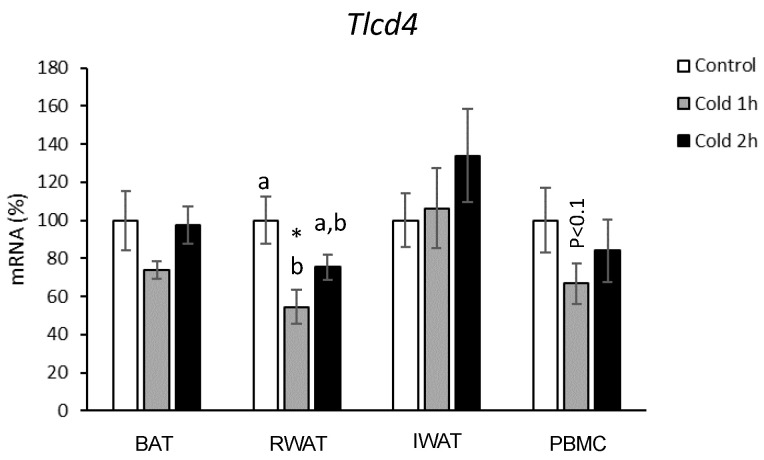
*Tlcd4* gene expression in BAT, retroperitoneal (RWAT), inguinal WAT (IAT), and PBMCs of female rats housed at different room temperatures: 22 °C (Control) or 4 °C for 1 (Cold 1 h) or 2 h (Cold 2 h). mRNA expression was measured by real-time RT-qPCR. Results represent means ± SEM (*n* = 6–8) of ratios of specific mRNA levels relative to *Lrp10* or *Gdi*, expressed as a percentage, where the control group was set to 100%. Statistics: the * symbol shows the significance of the cold-exposed groups vs. the control group (U Mann–Whitney, *p* < 0.05, or indicated when different). Bars not sharing common letters (a, b) are significantly different (one-way ANOVA, *p* < 0.05). DMS post hoc was used after one-way ANOVA analysis.

Because longer-term cold exposure is not feasibly translated to humans, we also performed an experiment that mimics the human study, with acute cold exposure. As shown in Figure 3, one-hour cold exposure resulted in a down-regulation of *Tlcd4* mRNA levels in retroperitoneal WAT (One-way ANOVA, *p* < 0.05). A trend in down-regulation for this gene was also observed in the PBMCs of these animals (Mann–Whitney U test, *p* = 0.07). However, two hours of cold exposure did not reveal effects on the *Tlcd4* gene expression, neither in the adipose depots or in the PBMCs.

**Figure 4:**
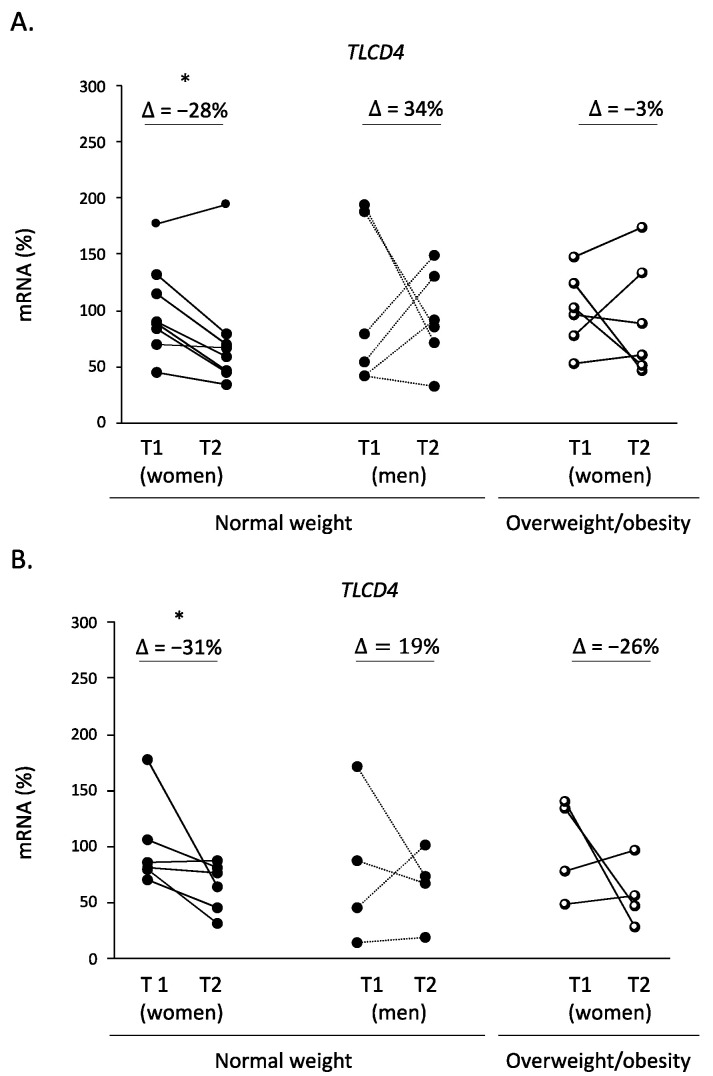
Effects of cold exposure on *TLCD4* gene expression in PBMCs of women and men with normal weight, and women with overweight or obesity. *TLCD4* mRNA expression was measured by real-time RT-qPCR in PBMC samples collected 4 h (T1) and 6 h (T2) after feeding, with (**A**) or without (**B**) cold exposure in this timelapse. Results represent means ± SEM (*n* = 4–8) of ratios of specific mRNA levels relative to *RPLP0*, expressed as a percentage, where T1 was set as 100%. Statistics: the * symbol shows the significance of the cold-exposed groups vs. the control group (Wilcoxon test, *p* < 0.05).

We also analyzed the *TLCD4* expression in humans to validate its usefulness as a cold-exposure biomarker. In PBMCs, the *TLCD4* gene expression showed a 28% decrease with two-hour acute cold exposure in women with normal weight, but not in those with overweight or in normal-weight men (Wilcoxon test, *p* < 0.05) (Figure 4). However, this decreased expression observed for *TLCD4* in the PBMCs of normal-weight women was also evident when participants were not cold-exposed. This points to the fact that *TLCD4* expression could be affected by the post-absorptive fasting state; participants had breakfast at 7:00 a.m., and PBMC samples were collected 4 and 6 h later (with or without cold exposure in this timelapse) (Figure 4).

## 4. Discussion

Responsiveness to cold can currently only be established using fluoro-glucose in a PET scan, which requires specific expertise and expensive equipment. This limits the research possibilities. To resolve this, we aimed to identify biomarkers reflecting WAT/BAT cold exposure, preferably in an easily obtainable biological material such as PBMCs. Using ferret, rat, and human samples, we identified *TLCD4* as a potential biomarker for AT cold exposure, reflected in the PBMCs.

In the widely used rodents, the thermogenic response to cold primarily occurs in BAT, which also shows WAT browning, but to a smaller extent. Humans, however, do not have a well-defined BAT, and the metabolic effects of cold importantly involve WAT browning [24]. Therefore, we first focused on ferrets, with an AT morphology closer to humans. Here, using microarray analysis, we describe that one-week cold exposure had a common effect on gene expression in two different adipose tissues of ferrets, the IAT and the aPVAT, as well as in PBMCs. In general, as a result of cold exposure, we observed a down-regulation in the gene expression of regulatory transcription factors (especially zinc finger protein-coding genes), a down-regulation in genes related to extra- and intracellular structure and cell cycle, and an up-regulation of genes related to inflammation and antigen recognition, though we have previously described a generalized anti-inflammatory gene expression profile in the PBMCs and aPVAT of cold-exposed ferrets [22]. Three of the genes affected by cold exposure are involved in lipid homeostasis/metabolism, *Soat1*, *Them4*, and *Tlcd4*, and all of them were up-regulated. *Tlcd4* is especially relevant, as it was the unique gene equally regulated in the two studied adipose tissues and in PBMCs when using a more restrictive *p*-value (Student’s *t*-test, *p* < 0.01). Using the same set of ferrets, we have already revealed that cold exposure induces a clear white-to-brown remodeling of aPVAT, with the appearance of multilocular adipocytes highly stained for UCP1 [25]. Thus, the increased expression of *Tlcd4* observed not only in aPVAT but also in IAT and PBMCs, could be considered a general marker of cold exposure, not restricted to just a specific adipose tissue depot, in these animals. 

*Tlcd4* encodes for *TLC domain containing 4* (also known as *Transmembrane protein 56*, *Tmem56*), and there is hardly any available information regarding its function. It is predicted to be a membrane-bound protein predicted to be involved in lipid homeostasis [26]. The available information on *Tlcd4* reveals that its mRNA levels could be considered a single biomarker or as part of a set of transcripts of certain diseases, such as acute myeloid leukemia [27], hypertrophic obstructive cardiomyopathy [28], coronary heart disease [29], hypertension [30], acute respiratory distress syndrome induced by sepsis [31], or as a marker of the combined IFN-α therapy of viral hepatitis C [32]. Here, we describe for the first time the relationship between the expression of this gene and cold exposure. 

To confirm the usefulness of *Tlcd4* for cold exposure research, we analyzed its expression in different adipose tissue depots of rats of different ages. Interestingly, the same response (increased expression) observed in ferrets was evident in BAT of 4-month-old female rats after one week of cold exposure. Previously published data in this set of rats showed that cold exposure induced an up-regulation of *Pgc1a* and *Fgf21*, which are good markers of cold-activated BAT [12]. PBMCs also revealed an increased *Tlcd4* expression at the age of 4 months, in concordance with the previously reported increased expressions of other well-defined brown markers in these cells in the same animals [12]. All in all, *Tlcd4* transcript levels could be considered a suitable biomarker of cold exposure in adipose tissue, which can also be analyzed in PBMCs. However, *Tlcd4* expression seems to be modulated by the duration of cold exposure, because contrary to what was observed with longer-term cold exposure (one week), an acute cold exposure of one hour resulted in decreased *Tlcd4* mRNA levels in the retroperitoneal WAT of one-month-old female rats, and the same profile was observed in PBMCs. This time-dependent (acute/chronic) response to cold has already been previously described in piglets: acute cold exposure (4 °C for 10 h) induced the expression of several brite/brown adipocyte markers, while longer-term cold exposure (15 days) induced the down-regulation of these markers, e.g., *Ucp1* and *Prdm16* in subcutaneous AT [33]. 

PBMCs can reflect gene expression patterns occurring in other internal tissues, such as adipose tissue, and is therefore widely used for the search for non-invasive biomarkers [34]. It has been previously described that human PBMC express brown/brite markers and the expression of a subset of the analyzed target genes is affected by cold exposure [14]. Thus, this easily obtainable biological material can be used to perform cold research with minimum invasiveness. Here, we show that acute cold exposure (2 h) reduced PBMC *TLCD4* mRNA levels in women with normal weight. However, the same profile was evident for *TLCD4* gene expression when participants were not cold-exposed. Thus, the observed effect may be attributed to the short-term post-absorptive fasting during the methodological procedure. Since the exact function of *TLCD4* is not well defined, our data are of interest as they point to a potential role of this gene on fasting-related fat mobilization. On the other hand, *TLCD4* expression was not affected either by cold exposure or post-absorptive fasting in women with overweight/obesity. This lack of response is in accordance with our previous results showing fasting insensitivity in rodents with overweight/obesity reflected at the gene expression level in PBMCs (reviewed in [34]). In addition, despite some discrepancies [35], different studies have revealed that cold-exposure thermogenic activation is blunted in subjects with overweight [18,36]. On the other hand, *TLCD4* expression was not affected in normal-weight men. This sex difference could be due to the known difference in response to fasting, being greater in women vs. men [37,38]. In the same way, it is known that men present a lower thermogenic potential vs. women at adult age (as reviewed in [16]). Remarkably, cold exposure did not induce greater *TLCD4* down-regulation in PBMCs to the effect that could be attributed to short-term fasting. This could be due to the fact that a lower temperature is required to further decrease the expression of this gene. Finally, we cannot exclude that our (lack of significant) results could be due to the interindividual variability. This variability was recently shown by applying a temperature increase and decrease to human participants in order to analyze the thermoneutral zone and its lower critical point [39]. 

In conclusion, we provide the first evidence pointing to *Tlcd4* modulation by cold exposure. Thus, its gene expression analysis may be useful as a marker to assess the effect of cold, which has been validated in two animal models (ferrets and rodents). This biomarker could reflect thermogenic activation in response to acute and longer-term cold exposure. The results in humans are less clear and require further analysis, though our results show that *TLCD4* expression in human PBMC could be modulated by short-term fasting. The potential of *Tlcd4* gene expression as a cold exposure/fat mobilization biomarker is especially relevant, as it is not only detectable in different adipose tissues but also in an easily obtainable biological material, such as PBMCs in blood.

## Data Availability

The original contributions presented in the study are included in the article/Supplementary Materials, further inquiries can be directed to the corresponding author.

## Supplementary Materials

The following supporting information can be downloaded at https://www.mdpi.com/article/10.3390/biom14080935/s1: Table S1: Age and anthropometrical parameters of the human study: body weight and height, BMI, body fat, waist perimeter, and hip perimeter. Results represent means ± SEM (n = 6–8); Table S2: Nucleotide sequences of primers and amplicon size used for real-time RT-qPCR amplification.
