# Supplementary material for: TLCD4 as Potential Transcriptomic Biomarker of Cold Exposure
Source: Biomolecules. 2024 Aug 1;14(8):935. doi: 10.3390/biom14080935 (PMC11352221; doi:10.3390/biom14080935)
Supplement: Supplementary file 1 [file biomolecules-14-00935-s001.zip › Table S1.pdf]

Table S1.

| | Age (years) | Body weight (g) | Body height (cm) | BMI (%) | Body fat (%) | Waist perimeter (cm) | Hip perimeter (cm) |
|---|---|---|---|---|---|---|---|
| Women with normal-weight | 21.9 ± 1.0 | 58.4 ± 2.7 | 167 ± 2 | 21.0 ± 0.7 | 31.8 ± 1.0 | 68.1 ± 1.8 | 96.3 ± 1.8 |
| Men with normal-weight | 22.5 ± 1.6 | 73.3 ± 4.1 | 180 ± 4 | 22.5 ± 0.7 | 17.9 ± 2.3 | 80.3 ± 3.4 | 100 ± 3 |
| Women with overweight or obesity | 23.8 ± 2.3 | 77.9 ± 4.7 | 159 ± 3 | 31.1 ± 2.2 | 40.6 ± 2.4 | 83.0 ± 4.8 | 113 ± 5 |
