# Supplementary material for: TLCD4 as Potential Transcriptomic Biomarker of Cold Exposure
Source: Biomolecules. 2024 Aug 1;14(8):935. doi: 10.3390/biom14080935 (PMC11352221; doi:10.3390/biom14080935)
Supplement: Supplementary file 1 [file biomolecules-14-00935-s001.zip › Table S2.pdf]

Table S2.

	Forward	Reverse	Amplicon size (pb)
TMEM56			
Rat	agcaccgagaagaagatcga	aggtagcctgaagcagtcga	173
Ferret	tggacaccaacaccaaactg	aagcccaaccaccaaagaa	191
Human	tgccattctttggtggttg	catacagggacgcacaatga	214
**Reference Genes**			
Gdi (for rat)	ccgcacaaggcaaatacatc	gactctctgaaccgtcatcaa	159
Lrp10 (for rat)	tcccctttcttctcctcctc	ttaccgtctgttccttgctg	198
Mettl2b (for ferret)	ggtcgttccagacaagatgc	ccgtcccctctcacatagaa	162
RPLP0 (for human)	acaacccagctctggagaaa	tgcccctggagattttagtg	240
